# Root-Derived *Flammulina velutipes* Polysaccharides Improve Myofibrillar Protein Stability and Maintain *Catfish* Surimi Quality During Freeze–Thaw Cycling

**DOI:** 10.3390/gels12040285

**Published:** 2026-03-28

**Authors:** Ruiying Chen, Ning He, Xiaodong Li, Yu Zhan, Xin Zhang, Yingchun Zhu

**Affiliations:** 1 College of Food Science and Engineering, Shanxi Agricultural University, Jinzhong 030801, China; 18734422667@163.com (R.C.); 18335463905@163.com (N.H.); lixiaodong0023@163.com (X.L.); 13488964920@163.com (Y.Z.); 2 Datong Yunzhou District Yellow Flower Industry Development Service Center, Datong 037303, China; zhangxin980523@163.com

**Keywords:** *catfish* surimi, flammulina velutipes polysaccharides, myofibrillar protein, cryoprotective, freeze–thaw cycle, gel quality

## Abstract

Frozen surimi, a commonly used raw material in processed aquatic products, is vulnerable to repeated freeze–thaw fluctuations that accelerate protein denaturation and quality loss. In this study, root-derived *Flammulina velutipes* polysaccharides (FVPs) were extracted from the root-like portion of enoki mushroom, and surimi supplemented with 2% FVP and a blank control (CK) were stored at −18 °C and subjected to a total of five freeze–thaw cycles. The effects of FVP on myofibrillar protein (MP) characteristics and the storage quality of *catfish* surimi during the freeze–thaw cycles were analyzed. Compared with CK, FVP markedly alleviated the deterioration of water-holding capacity, gel strength, and MP solubility throughout freeze–thaw cycling. It also effectively inhibited the increase in thiobarbituric acid reactive substance (TBARS) values and MP aggregation and delayed the rate of decrease in the storage modulus (G′) and loss modulus (G″) of surimi. Additionally, low-field nuclear magnetic resonance (LF-NMR) further showed that FVP limited the conversion of immobilized water to free water, indicating enhanced water retention under repeated freeze–thaw stress. Fourier transform infrared spectroscopy (FTIR) and scanning electron microscopy (SEM) analyses revealed that FVP stabilized the secondary structure of MPs, making the microstructure of surimi more uniform and compact. The results of this study indicate that FVP exhibited significant cryoprotective effects during freeze–thaw cycles of surimi relative to the untreated control group, providing a theoretical basis for its potential application in aquatic product storage.

## 1. Introduction

Surimi, an important aquatic processed product, is widely consumed worldwide because of its high nutritional value, distinctive flavor, and favorable processing performance [1]. To ensure year-round availability and meet the demands of long-distance trade, frozen storage has been adopted as one of the primary preservation approaches for surimi products [2]. However, unavoidable temperature fluctuations during cold-chain distribution and household storage can lead to the repeated thawing and refreezing of frozen foods. Such temperature-variation-induced freeze–thaw (F-T) cycling has been reported to markedly impair the texture and gel-forming properties of surimi [3,4]. During F-T cycling, repeated temperature variation promotes ice recrystallization, and the progressive growth of ice crystals exerts mechanical compression on muscle cells, thereby disrupting the integrity of cellular membranes [5]. This structural damage not only compromises the myofibrillar protein (MP) network [6] but also disrupts cellular compartmentalization, thereby accelerating oxidative reactions. Previous studies have demonstrated that repeated F-T cycling intensifies the oxidative modification of protein side chains, as evidenced by decreased sulfhydryl content and the accumulation of carbonyl derivatives, along with a significant increase in lipid oxidation products such as malondialdehyde [7]. Oxidation-induced protein unfolding and aggregation further weaken gel formation, leading to moisture loss, nutrient depletion, and the deterioration of the overall functional quality, ultimately limiting the commercial value and consumer acceptance of surimi products [8,9].

To mitigate freeze–thaw damage, the incorporation of cryoprotectants has been widely employed as an effective industrial strategy. Although conventional commercial cryoprotectants (e.g., sugars and phosphates) can be beneficial, their application is constrained by excessive sweetness, potential health concerns (e.g., high phosphorus intake), and poor compatibility with clean-label requirements [6,10]. Therefore, the development of efficient, natural, and safe biopolymers as novel cryoprotectants has become a major research focus in aquatic food processing. Polysaccharides, owing to their strong hydrophilicity, steric hindrance effects, and ability to interact with proteins and water molecules through hydrogen bonding, have shown considerable promise in enhancing the quality of frozen foods [11,12]. For example, Cao et al. [13] found that inulin addition altered the water distribution in surimi, lowered its freezing point, inhibited ice crystal growth, and enhanced protein–protein interactions. Similarly, Gao et al. [14] demonstrated that soluble soybean polysaccharides effectively alleviated MP denaturation and structural disruption in silver carp surimi.

*Flammulina velutipes*, a common edible and medicinal fungus, is rich in *Flammulina velutipes* polysaccharides (FVPs). Previous studies have demonstrated that FVP exhibits multiple bioactivities, including antioxidant and immunomodulatory effects [15,16], and also provides desirable thickening, stabilizing, and gelling functionalities in food systems [17]. In our previous study, 2% FVP provided a level of protein protection in *catfish* surimi during 75-day storage at −18 °C that was comparable to that achieved with a conventional sucrose-sorbitol cryoprotectant (4% + 4%) [18]. However, systematic and in-depth evidence remains limited regarding how FVP performs under more complex and highly destructive freeze–thaw cycling, particularly in terms of MP structural and functional properties, water migration behavior, and overall surimi quality.

In light of these considerations, this study aimed to promote the value-added utilization of food-processing by-products. To this end, root-derived *Flammulina velutipes* polysaccharides—a fraction often discarded during processing due to the firm, fibrous texture of the basal portion—were extracted and incorporated into *catfish* surimi to examine their effects on surimi quality and myofibrillar protein (MP) characteristics under repeated freeze–thaw cycling. Multiple indices were determined, including water-holding capacity, gel strength, protein solubility, lipid oxidation, protein aggregation state, rheological properties, water distribution and migration, protein secondary structure, and microstructural characteristics, to elucidate the protective role of FVP and the potential molecular mechanisms underlying its action during repeated freeze–thaw treatments. To the best of our knowledge, this is the first study to systematically clarify the cryoprotective mechanism of root-derived *Flammulina velutipes* polysaccharides on the MPs in surimi during freeze–thaw cycling, with a particular emphasis on the dynamics of water migration and the stability of protein secondary structure. Together, these results offer mechanistic insights and experimental support for using FVP as a natural cryoprotectant in aquatic products.

## 2. Results and Discussion

### 2.1. Effects of FVP on MP Characteristics in Surimi During Freeze–Thaw Cycling

#### 2.1.1. Solubility

**Figure 1:**
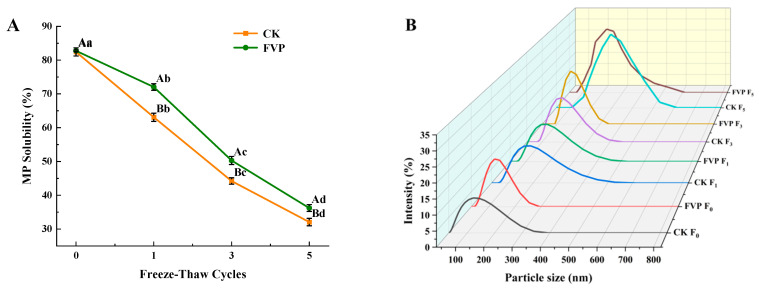
Effect of FVP on solubility (**A**) and particle size (**B**) of MP in surimi during freeze–thaw cycles. CK: control group; FVP: 2% FVP. Different uppercase letters indicate significant differences (*p* < 0.05) among treatments at the same freeze–thaw cycle, while different lowercase letters indicate significant differences (*p* < 0.05) among freeze–thaw cycles within the same treatment.

MP solubility is widely used to evaluate the physicochemical properties of MPs and is closely linked to their functionality [19]. Figure 1A shows the effects of FVP on MP solubility during freeze–thaw cycling. As shown, the MP solubility of fresh surimi was 82.43%. With additional freeze–thaw cycles, solubility decreased significantly (*p* < 0.05); after five cycles, MP solubility declined by 46.52% and 56.22% in the FVP and CK groups, respectively. Consistent results were reported for snakehead fish (Channa argus), where MP solubility declined significantly as the number of freeze–thaw cycles increased [20]. Ice crystal formation during freeze–thaw cycling can disrupt the spatial conformation of surimi proteins and is often accompanied by protein oxidation and denaturation, as well as the formation of disulfide and hydrophobic interactions. These changes weaken protein–water interactions, thereby lowering solubility and accelerating quality deterioration [21]. Throughout the entire freeze–thaw process, MP solubility remained higher in the FVP-treated samples than in the CK group (*p* < 0.05). This effect was attributed to the abundant hydroxyl groups in FVP, which can associate with MP molecules and promote a more hydrated state, thereby helping to alleviate aggregation-related changes in MPs and reduce the loss of solubility [18].

#### 2.1.2. Particle Size

Particle size is commonly used as a proxy for MP aggregation because oxidative modification can trigger intermolecular cross-linking and cluster formation, which typically enlarges protein particles [22]. Figure 1B presents the effects of FVP on MP particle size during freeze–thaw cycling. After freeze–thaw treatments, the particle-size distribution of MP solutions shifted toward larger sizes. This shift was likely caused by freeze-induced structural alterations and ice crystal damage, which increase protein–protein contacts and strengthen intermolecular associations, including hydrophobic interactions as well as hydrogen, disulfide, and ionic bonding [23]. Compared with the CK group, the FVP group consistently exhibited smaller particle sizes. Notably, by the fifth cycle, the mean particle size was 220 nm in the FVP group versus 295 nm in the CK group, suggesting that FVP may help maintain a more uniform MP dispersion and alleviate freeze–thaw-induced aggregation. However, since the above-mentioned indicators, namely, solubility and particle size, are insufficient to fully characterize protein denaturation, the protective effect of FVP on MP stability requires further verification using direct protein denaturation markers, such as Ca^2+^-ATPase activity and sulfhydryl group content.

#### 2.1.3. Rheological Properties

**Figure 2:**
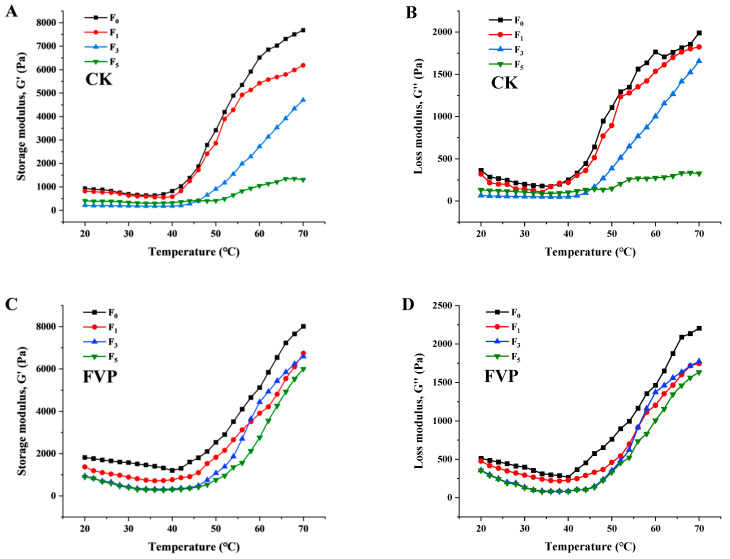
Effect of FVP on rheological properties of surimi during freeze–thaw cycles. (**A**,**C**) Storage modulus (G′); (**B**,**D**) loss modulus (G″). CK: control group; FVP: 2% FVP.

The storage modulus (G′) is commonly used to characterize the elastic component associated with MP network formation, while G″ (loss modulus) represents the viscous dissipation of the system [24]. The temperature-dependent evolution of G′ and G″ for surimi with and without FVP during freeze–thaw cycling is presented in Figure 2. Overall, similar variation trends in G′ and G″ were observed for both the CK and FVP groups. A decrease in both G′ and G″ was observed below 40 °C. This stage is associated with the denaturation of the myosin tail region and the dissociation of α-helical structures, during which intermolecular chemical bonds are formed and the gel network is gradually initiated [25]. Once the temperature exceeded 40 °C, both G′ and G″ began to increase. This increase was associated with the enhanced activity of endogenous proteolytic enzymes, which are generally responsible for this disruptive effect and can disrupt the preformed network; promote molecular unfolding/extension; and increase the mobility of the surimi gel matrix, with a relatively slow rate of increase observed [26]. When the temperature was raised above 50 °C, both moduli rose sharply, consistent with the intensified hydrophobic interactions and disulfide-bond formation that consolidate an irreversible and ordered gel network.

As shown in Figure 2, fresh surimi exhibited relatively high G′ and G″ values, and both parameters were further increased by FVP addition, indicating that FVP enhanced the viscoelasticity of surimi gels. With increasing numbers of freeze–thaw cycles, G′ and G″ decreased in both groups, likely because repeated freeze–thaw treatments induced MP denaturation, weakened intermolecular cross-linking, and reduced gelation capacity. Within the same temperature range, all samples followed a similar trajectory, with G′ and G″ decreasing initially and then increasing upon further heating. However, the terminal values of both moduli dropped progressively as freeze–thaw cycles accumulated in both the CK and FVP groups. Notably, after five cycles, the final G′ and G″ values of the CK group (1307.5 and 326.15 Pa, respectively) were markedly lower than those of the FVP group (6000.12 and 1632.40 Pa, respectively). These results indicate that the elasticity of surimi gels gradually decreased with increasing freeze–thaw cycles. However, FVP addition alleviated this deterioration, probably because FVP interacted with MP molecules through hydrogen bonding and physical entanglement, thereby stabilizing the gel network and enhancing its viscoelastic properties [18]. Consistently, Zhu et al. [27] reported that during frozen storage (−18 °C, 60 d), both carboxymethyl chitosan (CMCh) and a commercial cryoprotectant (4% sucrose + 4% sorbitol) effectively slowed the reductions in G′ and G″ in surimi systems and maintained an elasticity-dominant gel behavior; notably, CMCh exhibited superior performance in preserving gel-network stability and rheological structure during mid-to-late storage.

#### 2.1.4. Secondary Structure

Protein peptide bonds exhibit several characteristic vibrational bands, including the amide I band (1600–1700 cm^−1^), amide II band (1550–1600 cm^−1^), and amide III band (1200–1400 cm^−1^). Among these, the amide I band is the most sensitive region for evaluating protein secondary structure. Accordingly, secondary structure alterations are commonly interpreted from the relative proportions of α-helix, β-turn, β-sheet, and random coil [14]. The representative deconvolution of the amide I region and the corresponding FTIR spectra of the CK and FVP groups are presented in Supplementary Figures S1 and S2, respectively. Table 1 summarizes the variations in the MP secondary structure components (α-helix, β-turn, β-sheet, and random coil) during freeze–thaw cycling.

As shown in Table 1, β-turn constituted the largest fraction of the secondary structure in fresh *catfish* MPs (28.77%), followed by β-sheet (25.99%) and α-helix (24.54%), whereas random coil accounted for a smaller proportion (20.67%). After FVP addition, the proportions of α-helix and β-turn increased, accompanied by a reduction in β-sheet content. Because α-helical structures have been reported to play an important role in stabilizing MPs [28], the higher α-helix content observed in the FVP group than in the CK group provides a plausible explanation for the increased gel strength following FVP incorporation. In addition, the representative deconvolution result suggests that the addition of FVP may affect the profile of the amide I band, indicating that non-covalent interactions such as hydrogen bonding may exist between FVP and MP.

As freeze–thaw cycles accumulated, α-helix and β-turn contents declined, while β-sheet and random coil fractions increased, indicating a progressive rearrangement of the MP secondary structure and a loss of conformational stability. After five cycles, α-helix content dropped from 24.54% to 18.89% in the CK group, whereas a smaller decrease was observed in the FVP group (25.45% to 20.43%), implying that FVP mitigated the freeze–thaw-induced disruption of MP secondary structure integrity. These structural changes may be associated with the exposure of hydrophobic groups and conformational alterations in the myosin head during freeze–thaw cycling, which promote α-helix unfolding and facilitate polypeptide-chain rearrangement into β-sheet structures. Peng, Li, Wang, et al. [29] reported a similar trend when investigating the effects of whey protein hydrolysates on the MP gel structure of Spanish mackerel surimi during freeze–thaw cycling: across seven cycles, α-helix and β-turn contents declined, accompanied by increases in β-sheet and random coil fractions.

### 2.2. Effects of FVP on Surimi Quality During Freeze–Thaw Cycling

#### 2.2.1. Gel Strength

**Figure 3:**
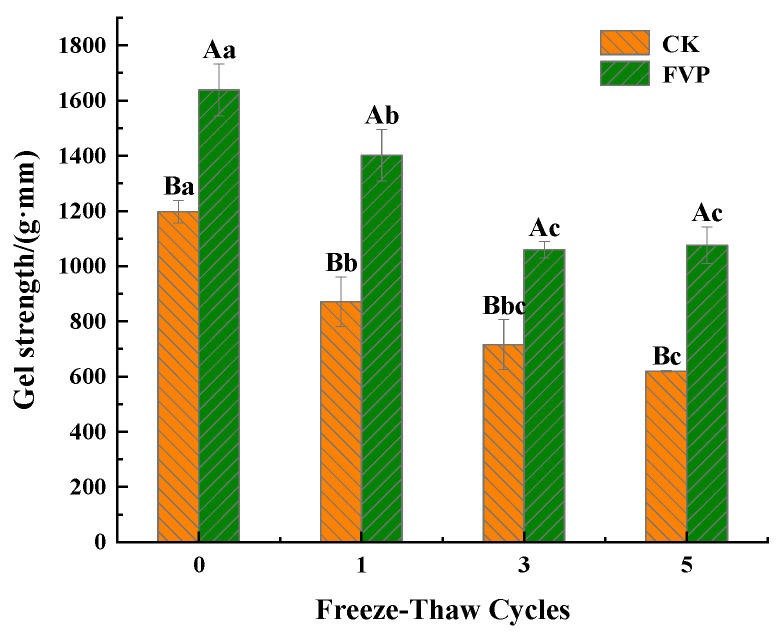
Effect of FVP on MP gel strength during freeze–thaw cycles. CK: control group; FVP: 2% FVP. Different uppercase letters indicate significant differences (*p* < 0.05) among treatments at the same freeze–thaw cycle, while different lowercase letters indicate significant differences (*p* < 0.05) among freeze–thaw cycles within the same treatment.

Gel strength is widely used to characterize surimi gel quality, as it is closely related to the textural perception and functional performance of surimi-based products [30]. Figure 3 shows the effects of FVP on gel strength during freeze–thaw cycling. In fresh samples, gel strength was 1197.67 g·mm in the CK group and increased to 1638.47 g·mm after FVP addition, corresponding to a 36.80% enhancement. Surimi gelation is a heat-induced gelling process that forms a continuous protein–water cross-linked network. When FVP is incorporated, a polysaccharide–protein–water ternary system is formed during heating. Yan et al. [31] similarly suggested that in polysaccharide–protein–water ternary systems, MP chains are more prone to rearrangement and molecular extension, which strengthens hydrophobic associations and favors the formation of a more compact gel network.

After five freeze–thaw cycles, the gel strength in the CK group dropped significantly, from 1197.67 to 619.64 g·mm (*p* < 0.05). In contrast, the gel strength in the FVP group declined from 1638.47 to 1075.39 g·mm. In general, the gel strength declined with successive freeze–thaw cycles, likely due to the cumulative and irreversible disruption of the gel network arising from repeated ice crystal formation and thawing. In addition, moisture loss and the enlargement of voids within the gel matrix during freeze–thaw treatment can further weaken gel strength [32]. Across all freeze–thaw stages, the FVP-treated samples consistently exhibited higher gel strength than the CK group (*p* < 0.05). Notably, after the fifth cycle, the gel strength of the FVP-treated gels was approximately 1.73-fold that of the CK group. This suggests that FVP helped maintain a tighter gel network and mitigated the freeze–thaw-driven loss of gel strength. Similarly, Wang et al. [33] reported that chitosan incorporation not only exerts pressure on the protein matrix through water absorption and swelling, but also promotes polysaccharide–protein associations through hydrogen bonding, functioning as an intermolecular “bridge” that limits the loss of gel strength. Ma et al. [34] further demonstrated that Naematelia aurantialba polysaccharides (NAPs) reduced the free-water fraction in tilapia surimi during frozen storage through their hygroscopicity and promoted protein cross-linking. Meanwhile, NAP was uniformly dispersed in the gel, helping maintain gel-network stability. The inhibitory effect of NAP increased with increasing addition level and consistently outperformed a commercial cryoprotectant (4% sucrose + 4% sorbitol).

#### 2.2.2. Microstructure

**Figure 4:**
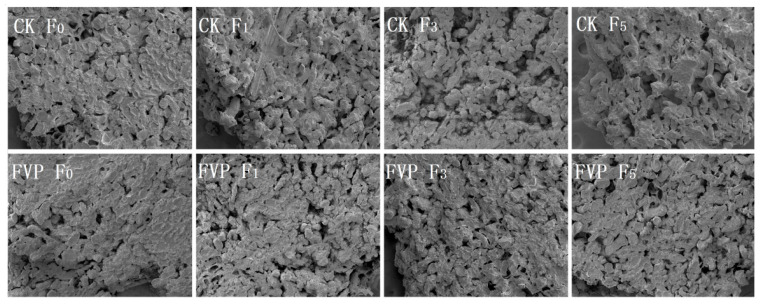
The effect of FVP on the microstructure of MP gels during freeze–thaw cycles. CK: control group; FVP: 2% FVP.

The evolution of the gel microstructure across freeze–thaw cycles is presented in Figure 4. At zero cycles, both the CK and FVP groups exhibited a gel matrix with a relatively smooth surface, small pores, and a compact structure. With increasing freeze–thaw cycles, the pore size gradually increased in both groups; however, the pores in the CK gels were consistently larger than those in the FVP gels. After three cycles, the CK gel structure became visibly loose and porous, whereas the FVP gels still maintained smaller, more uniformly distributed pores. After five cycles, the CK gels displayed a rough surface with further enlarged and irregular pores. In contrast, the FVP gels remained comparatively compact and intact. These observations indicate that freeze–thaw cycling exerts substantial destructive effects on the gel microstructure, and the extent of deterioration becomes more pronounced with increasing cycle numbers [32].

With FVP incorporation, the gel surface appeared flatter and the network was more uniform and compact than that of the CK group. This improvement was attributed to the ability of FVP to act as a filler that penetrates the gel network, strengthens interactions with MPs, and suppresses freeze–thaw-induced MP denaturation and aggregation. In addition, freeze–thaw damage is closely associated with the formation and recrystallization of ice crystals, which may mechanically disrupt the gel network and enlarge pores, thereby aggravating microstructural deterioration during freeze–thaw cycling. The better-preserved microstructure observed in the FVP group suggests that FVP may alleviate the structural damage associated with ice crystal growth and thus help maintain the integrity of the gel network. However, ice crystal morphology was not directly characterized in the present study. Therefore, further studies on ice crystal size, distribution, and recrystallization behavior are required to verify the proposed cryoprotective mechanism of FVP. Collectively, these results suggest that FVP provides structural protection for surimi gels during freeze–thaw cycling. This observation aligns with the findings of Wang et al. [30], in which grass carp surimi supplemented with pomegranate peel polysaccharides (PPPs) retained a dense gel network with smaller pores even after five freeze–thaw cycles, further supporting the protective role of polysaccharides in preserving surimi gel microstructure under freeze–thaw conditions.

#### 2.2.3. Water-Holding Capacity

**Figure 5:**
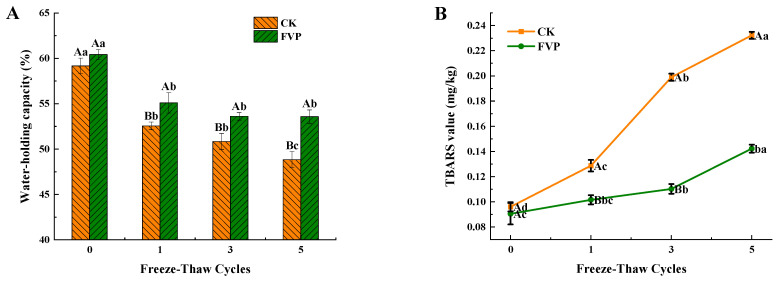
Effect of FVP on water-holding capacity (**A**) and TBARS values (**B**) during freeze–thaw cycles. CK: control group; FVP: 2% FVP. Different uppercase letters indicate significant differences (*p* < 0.05) among treatments at the same freeze–thaw cycle, while different lowercase letters indicate significant differences (*p* < 0.05) among freeze–thaw cycles within the same treatment.

Water-holding capacity (WHC) is commonly used to assess the quality of surimi because it reflects network integrity and the extent of protein denaturation [35]. As presented in Figure 5A, fresh surimi showed comparable WHC values in the CK (59.18%) and FVP (60.42%) groups (*p* > 0.05). With progressive freeze–thaw cycling, WHC exhibited an overall decreasing trend, which can be attributed to repeated freeze–thaw-induced ice recrystallization and muscle-cell structural damage, ultimately leading to impaired water retention [36]. Across all cycles, the FVP-treated samples consistently maintained higher WHC than the CK group (*p* < 0.05). After the fifth cycle, WHC decreased to 48.83% in the CK group, whereas a higher WHC of 53.58% was maintained in the FVP group, representing a 4.75% absolute increase relative to CK. Importantly, no significant difference in WHC was observed for the FVP group between the third and fifth cycles (*p* > 0.05), while the CK group continued to decrease significantly, suggesting that the protective effect of FVP on WHC becomes particularly evident at the later stages of freeze–thaw cycling. Zhang et al. [37] similarly reported that carrageenan oligosaccharides (CGOs) improved intermolecular interactions within herring surimi gels, thereby effectively maintaining WHC during late-stage freeze–thaw treatment. This effect was mainly attributed to the hydroxyl groups in FVP, which can stabilize water molecules around proteins and inhibit ice recrystallization. Accordingly, the conversion of immobilized water to free water is suppressed, which helps attenuate the freeze–thaw-induced loss of WHC [38].

#### 2.2.4. TBARS

TBARS values are commonly used to evaluate the extent of lipid oxidation and rancidity in surimi during frozen storage; higher TBARS values indicate more severe lipid oxidation [39]. The evolution of TBARS values during freeze–thaw cycling is presented in Figure 5B. In fresh surimi, TBARS values were 0.08 mg/kg for the FVP group and 0.09 mg/kg for the CK group, showing no significant difference (*p* > 0.05) and remaining at low levels. As freeze–thaw cycling progressed, TBARS values increased in both treatments. This rise may be attributed to ice-crystal-induced structural disruption, which facilitates the release of pro-oxidant components and lipid-oxidation precursors, particularly free iron ions. These ions can participate in electron-transfer reactions with molecular oxygen, generating lipid peroxyl radicals/anions and thereby accelerating lipid oxidation and elevating TBARS values [5,40]. After the fifth cycle, the TBARS value in the CK group reached 0.23 mg/kg, approximately 2.56-fold that of the fresh sample. In contrast, the FVP group increased only to 0.14 mg/kg, corresponding to 1.75-fold of the fresh level, and remained significantly lower than the CK group (*p* < 0.05). These results indicate that FVP effectively inhibited oxidative deterioration during repeated freeze–thaw cycling. As a polysaccharide rich in hydroxyl groups, FVP can bind free water and promote its conversion to more tightly associated water, thereby lowering the system’s eutectic point temperature and reducing ice crystal formation during frozen storage. As a result, the progression of lipid oxidation in surimi can be slowed. Similar cryoprotective behavior has also been reported for other marine polysaccharides. Zhu et al. [41] showed that Sargassum fusiforme polysaccharide (SFP) effectively suppressed lipid oxidation in large yellow croaker filets during storage at −18 °C, with the strongest inhibitory effect observed during the middle and later stages of storage. When the TBARS value reached its peak on day 80, the value in the SFP group (0.12 mg/kg) remained markedly lower than those in the commercial cryoprotectant group (0.21 mg/kg) and the control group (0.35 mg/kg). Consistent with this trend, FVP in the present study maintained TBARS values at a relatively low level (0.14 mg/kg) even after five freeze–thaw cycles, suggesting that hydroxyl-rich polysaccharides may help alleviate lipid oxidation in frozen aquatic products.

#### 2.2.5. Water Distribution

**Figure 6:**
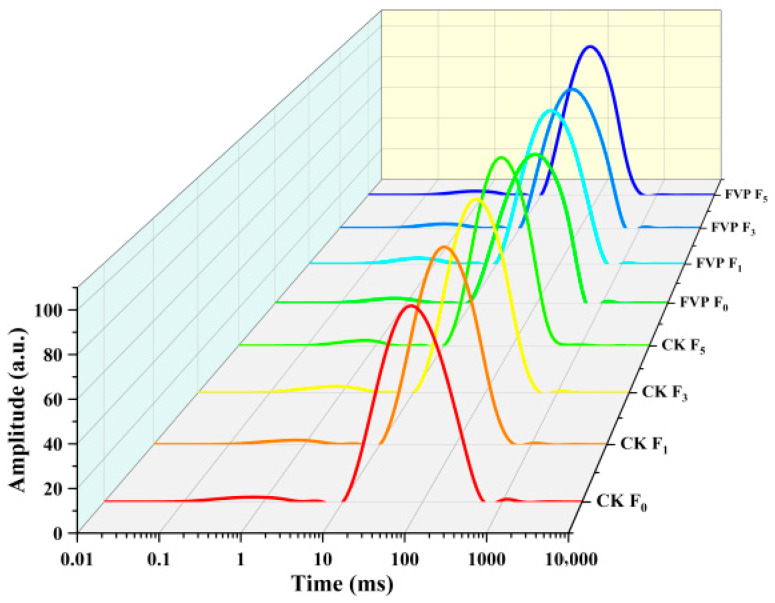
Effect of FVP on transverse relaxation time (T_2_) of surimi during freeze–thaw cycles. CK: control group; FVP: 2% FVP.

To better understand how FVP influences water status during freeze–thaw cycling, water populations in surimi were analyzed by LF-NMR. This technique tracks the transverse relaxation (T_2_) behavior of water protons and is widely applied to assess water-related physicochemical changes in surimi under frozen storage and distribution conditions [42]. Figure 6 presents the T_2_ relaxation spectra of *catfish* surimi. Two major peaks were observed at T_21_ and T_22_, corresponding to the proton spin-spin relaxation times of water. T_21_, the peak with the shortest relaxation time (0.72–3.05 ms), represents bound water tightly associated with proteins. T_22_, the dominant peak region with relaxation times of 104.70–110.92 ms, corresponds to immobilized (less mobile) water entrapped within the gel network. As freeze–thaw cycling progressed, the spectra exhibited a slight rightward shift, indicating that the water distribution in surimi was altered.

As shown in Table 2, immobilized water (T_22_) was the predominant water fraction in surimi, accounting for >97% of total water. This fraction is typically located between muscle fibers and is highly correlated with WHC. Bound water (T_21_) accounted for approximately 2% and is tightly associated with macromolecules, thus exhibiting the lowest mobility. Free water (T_23_) represented the smallest fraction and is readily lost under external forces, which is unfavorable for water retention. Table 2 shows that FVP shifted water populations toward immobilized and bound states, accompanied by a marked reduction in free water, indicating restrained water mobility/migration. With increasing freeze–thaw cycles, the proportion of immobilized water gradually decreased. After freeze–thaw treatment, the immobilized-water fraction decreased from 97.63% to 97.27% in the CK group and from 97.87% to 97.48% in the FVP group. Meanwhile, the bound-water fraction increased from 2.11% to 2.42% in the CK group and from 2.12% to 2.51% in the FVP group. Notably, the CK group showed an increase in free water (0.17% to 0.31%) during freeze–thaw cycling, while free water was undetectable in the FVP group across all stages, consistent with the high water-binding affinity of FVP. These results indicate that freeze–thaw cycling promoted the conversion of a portion of immobilized water into free water. This shift may be associated with freeze–thaw-induced changes in the MP secondary structure and decreased protein solubility, which can render the internal structure of surimi looser and more porous [43]. Li et al. [44] also reported that freeze–thaw-induced water redistribution and ice crystal formation are key drivers promoting the migration of immobilized water toward free water in surimi. The addition of FVP likely enhanced the association between water and MPs and restricted the conversion of immobilized water into free water. Zhang et al. [45] further found that polysaccharides can interact with ice crystals through hydrogen bonding, hydrophobic interactions, or electrostatic interactions, thereby influencing the distribution and mobility of the water molecules surrounding MPs and reducing the probability of ice recrystallization. Collectively, these findings suggest that FVP contributes to maintaining water stability in surimi during freeze–thaw cycling.

#### 2.2.6. Color

Surimi color is jointly determined by myoglobin content and the intrinsic color of added ingredients, and surimi with a whiter and more delicate appearance is generally preferred by consumers [43]. Table 3 summarizes the effects of FVP on surimi color parameters during freeze–thaw cycling. Relative to the CK group, FVP supplementation resulted in lower L* and whiteness values but higher a* and b* values (*p* < 0.05). Similar results were reported by Zhang et al. [46], who found that the addition of 1% FVP and 1% Pleurotus eryngii polysaccharide (PEP) increased the a* and b* values of silver carp surimi, accompanied by a decrease in whiteness. This effect was mainly attributed to the inherent yellow-brown hue of FVP, which darkened the surimi matrix and consequently reduced the L* value and whiteness. Although FVP incorporation decreased the whiteness of surimi to some extent, the growing consumer acceptance of clean-label products, together with the nutritional and functional benefits associated with FVP, may provide a basis for market acceptance of this color change. However, formal sensory evaluation is still needed to confirm its overall acceptability.

With increasing numbers of freeze–thaw cycles, lightness (L*) and whiteness continuously decreased in both the FVP and CK groups, whereas redness (a*) increased. A comparable pattern was reported by Li et al. [47], who examined Spanish mackerel (Scomberomorus niphonius) surimi supplemented with whey protein hydrolysates (WPHs; 0%, 5%, 10%, and 15%) under freeze–thaw cycling. Notably, in the FVP group, the changes in L* and whiteness from the first to the fifth cycle were not significant (*p* > 0.05), whereas the CK group exhibited a significant reduction in both L* and whiteness after the fifth cycle (*p* < 0.05). Compared with the CK group, surimi whiteness in the FVP group was therefore more stable throughout freeze–thaw cycling. To further characterize the magnitude of color variation during freeze–thaw treatment, the differences in lightness–darkness (ΔL*), redness–greenness (Δa*), blueness–yellowness (Δb*), and the overall color difference (ΔE*) were calculated based on the measured color parameters, and the results are summarized in Supplementary Table S1. The calculated ΔE* values showed that the CK group exhibited a slightly greater total color change than the FVP group after the first freeze–thaw cycle. Moreover, after five freeze–thaw cycles, the ΔE* value of the FVP group remained lower than that of the CK group, indicating that the incorporation of FVP helped mitigate overall color deterioration during repeated freeze–thaw treatments.

Color deterioration in surimi may be attributed to lipid oxidation and non-enzymatic reactions occurring during freeze–thaw treatment [48]. Hui et al. [49] reported that polysaccharides can scavenge reactive oxygen species (ROS) generated during lipid peroxidation, shorten the propagation chain length, and thereby interrupt or slow lipid peroxidation. In agreement with these observations, the present results suggest that *Flammulina velutipes* polysaccharides inhibited oxidative reactions in surimi, which contributed to improved color stability.

## 3. Conclusions

This work evaluated how FVP modulates the quality indices and MP-related characteristics of *catfish* surimi under repeated freeze–thaw cycling. The results demonstrated that supplementation with 2% FVP effectively alleviated the declines in water-holding capacity, gel strength, and rheological properties induced by freeze–thaw cycling. In addition, FVP suppressed the increase in TBARS values and alleviated freeze–thaw-induced changes in MP properties, as reflected by the higher MP solubility and smaller particle size in the FVP group. These effects were primarily attributed to the ability of FVP to maintain the MP secondary structure, enhance MP structural stability, and inhibit the conversion of immobilized water into free water during freeze–thaw treatment. Scanning electron microscopy further suggested that FVP incorporation contributed to a relatively denser microstructure with smaller pores in surimi gels.

Overall, these results suggest that FVP may help mitigate freeze–thaw-induced quality deterioration in *catfish* surimi under the present experimental conditions. In the absence of a commercial positive control, the relative cryoprotective efficacy of FVP compared with industry-standard cryoprotectants remains unknown. Therefore, further validation against commercial standards and additional mechanistic studies are needed to support its potential application in surimi systems.

## 4. Materials and Methods

### 4.1. Materials

*Flammulina velutipes* were obtained from the Edible Fungi Research Center, Shanxi Agricultural University. Eighty live African sharptooth *catfish* (*Clarias gariepinus)* (mean body weight, 1250 ± 50 g; mean body length, 30 ± 2 cm) were sourced from a local aquaculture market in Taoyuanbao Village, Taigu County. Sucrose and sorbitol were supplied by Shandong Dingsheng Chemical Co., Ltd., Jinan, China. Diethyl ether, KCl, HCl, NaCl, glycine, and EDTA were obtained from Chengdu Kelong Chemical Co., Ltd., Chengdu, China. Urea, DTNB, Coomassie Brilliant Blue, PIPES, sodium pyrophosphate, and Na_2_HPO_4_ were sourced from Tianjin Kaitong Reagent Co., Ltd., Tianjin, China. All chemicals were of analytical grade.

### 4.2. Preparation of Flammulina velutipes Polysaccharides (FVPs)

The basal (root-like) portion of *Flammulina velutipes* was excised, thoroughly washed, and dried in a drying oven at 70 °C for 24 h. The dried material was ground using a grinder (L-VP300; Hunan LAISDA Instrument Equipment Co., Ltd., Yueyang, China) for 30 s and passed through a 200-mesh sieve to obtain *Flammulina velutipes* powder. Ten grams of *Flammulina velutipes* powder was mixed with distilled water (1:30, *w*/*v*) and polysaccharides were extracted by ultrasonication at 70 °C (350 W) for 50 min. The resulting suspension was centrifuged at 8000 rpm for 10 min at 4 °C (5804R; Eppendorf, Hamburg, Germany), and the supernatant was concentrated to 20% of its initial volume at 55 °C using a rotary evaporator (RE-5286A; Shanghai Yarong, Shanghai, China). Ethanol precipitation was then performed by adding three volumes of anhydrous ethanol, followed by incubation at 4 °C overnight. The precipitate was recovered by centrifugation, pre-frozen at −78 °C, and freeze-dried for 48 h (ALPH1-2/LD-Plus; CHRIST, Osterode am Harz, Germany) to obtain FVP. The monosaccharide profile of FVP was determined by ion chromatography (ICS5000; Thermo Fisher Scientific, Waltham, MA, USA), revealing glucose (Glc), galactose (Gal), mannose (Man), fucose (Fuc), and xylose (Xyl) in a molar ratio of 55.5:25.9:10:5.5:2. The molecular weight distribution, measured by HPGPC, ranged from 4.946 to 9178.523 kDa.

### 4.3. Preparation of Catfish Surimi Samples

Fresh live *catfish* were transported to the laboratory under chilled conditions and held in ice water (0–4 °C) for 15 min prior to stunning by electric shock. Immediately after stunning, the fish were headed, eviscerated, deboned, and skinned to obtain large blocks of dorsal muscle. The filets were washed twice with 0.15% (*w*/*v*) NaCl solution (0–4 °C) and then drained.

The washed muscle was minced using a meat grinder and divided equally into two portions: a blank control and a treatment supplemented with 2% FVP (selected based on preliminary dose-screening experiments). Each portion was then chopped for 3 min in a bowl chopper (ZB-5; Shanghai Qingpu Food Packaging Machinery Factory, Shanghai, China) to prepare *catfish* surimi, while 20% (*w*/*w*) ice water was incorporated to maintain a low temperature. The two formulations were labeled CK and 2% FVP. The moisture content of surimi was determined using a vacuum drying oven (DZF-6050, Ruifeng Experimental Equipment Co., Ltd., Guangzhou, China) and controlled at 78 ± 1% (*w*/*w*). Surimi samples were portioned into polyethylene bags (thickness, 80 μm; size, 70 mm × 100 mm). The film’s oxygen and water vapor transmission rates at 25 °C were 300 cm^3^/(m^2^·24 h·0.1 MPa) and 2 g/(m^2^·24 h), respectively.

### 4.4. Freeze–Thaw Cycles (FTCs)

Freeze–thaw cycles (FTCs) were performed using the following protocol. The prepared surimi samples were stored at −18 °C for frozen storage. After 7 days, all samples were removed and thawed at 4 °C for 12 h. From each group, three packages were randomly taken for analysis, while the remaining samples were returned to −18 °C for continued storage; this sequence was regarded as one freeze–thaw cycle. Following this protocol, samples underwent 0, 1, 3, or 5 cycles, referred to as F_0_, F_1_, F_3_, and F_5_, respectively, after which the designated parameters were measured. This freeze–thaw regimen was designed to simulate the temperature fluctuations that may occur during cold-chain storage and household handling.

### 4.5. Extraction of Myofibrillar Protein (MP) and Determination of Protein Concentration

MP extraction was performed according to our previously reported procedure [50]. Briefly, 5.00 g of surimi was placed in a pre-chilled 100 mL centrifuge tube and combined with 20 mL of chilled extraction buffer (7.455 g/L KCl, 0.4066 g/L MgCl_2_, and 3.5814 g/L Na_2_HPO_4_; pH 7.0; ionic strength 0.19 mol/L). The mixture was homogenized for 30 s using a homogenizer (JH-ZS1, Hangzhou Jinghao Machinery Co., Ltd., Hangzhou, China) while kept in an ice bath to minimize oxidation and conformational changes. The homogenate was centrifuged at 2000× *g* for 15 min at 2 °C, and the supernatant was discarded. This extraction procedure was repeated three times. The pellet was then washed by resuspension in 40 mL of pre-chilled washing buffer (5.844 g/L NaCl; ionic strength 0.10 mol/L), followed by homogenization (30 s) and centrifugation (2000× *g*, 15 min, 2 °C). The washing step was conducted twice in total. The suspension was subsequently passed through two layers of gauze, and the filtrate was adjusted to pH 6.2 with cold 0.1 mol/L HCl. After centrifugation at 2000× *g* for 15 min at 2 °C, the supernatant was removed and the resulting pellet was collected as MP. The prepared MP was stored at 4 °C and used within 48 h. Protein content was quantified using the biuret assay with bovine serum albumin as the standard.

### 4.6. Determination of Myofibrillar Protein (MP) Characteristics in Surimi

#### 4.6.1. Solubility

A 5 mg/mL MP suspension (C_1_) was incubated at 2 °C for 4 h with intermittent shaking every 20 min. The mixture was then centrifuged at 2000× *g* for 15 min at 2 °C, and 1 mL of the supernatant (C_2_) was collected for protein determination. MP solubility was calculated using Equation (1):

(1) $$MP\;solubility\left(\%\right)=\frac{C_{2}}{C_{1}}\times 100\%.$$

#### 4.6.2. Particle Size

Particle size distribution of MP was determined by dynamic light scattering (DLS) using a Zetasizer Nano ZS90 (Malvern Instruments, Malvern, UK), following the method of Ye et al. [51]. Briefly, an MP dispersion (1 mg/mL) was analyzed at 25 °C after equilibration for 1 min, and the particle size distribution curves were obtained.

#### 4.6.3. Dynamic Rheological Analysis

Thawed surimi samples were analyzed on a rheometer (MCR 302; Anton Paar, Graz, Austria) equipped with a parallel-plate fixture (PP50) set to a 1 mm gap. Under oscillatory mode, a temperature sweep was performed within the linear viscoelastic region. The temperature was increased from 20 to 70 °C at a heating rate of 2 °C/min, with the oscillation frequency set at 1 Hz and the strain fixed at 0.1%.

#### 4.6.4. Secondary Structure Analysis

The MP secondary structure alterations were characterized by Fourier transform infrared spectroscopy (FTIR) following Xiao et al. [52]. Freeze-dried MP was mixed with KBr at a ratio of 1:100 (*w*/*w*), ground into a fine powder, and pressed into pellets. The spectra were recorded using a Tensor 27 FTIR spectrometer (Bruker Co., Ltd., Ettlingen, Germany) over the range of 4000–400 cm^−1^ with a spectral resolution of 4 cm^−1^. The amide I region (1600–1700 cm^−1^) was extracted, and the secondary structure components of MP were analyzed using PeakFit v4.12 software (Systat Software Inc., San Jose, CA, USA).

### 4.7. Determination of Quality-Related Properties of Surimi and MP Gels

#### 4.7.1. MP Gel Preparation and Gel Strength

MP gels were obtained by a two-stage heating protocol. Briefly, MP was dispersed in a buffer (containing 0.6 mol/L NaCl, pH 6.2), and the protein concentration was adjusted to 40 mg/mL. The MP solution was heated at 40 °C for 30 min and then at 80 °C for 30 min. After heating, the samples were cooled in an ice water bath for 30 min. The resulting MP gels were equilibrated at 4 °C for 24 h prior to analysis.

Gel strength was determined according to the method of Tian et al. [32]. The resulting gels were trimmed into 2 cm thick cylindrical specimens, and gel strength was determined using a texture analyzer (TA.XT Plus; Stable Micro Systems, Godalming, UK). Compression tests were performed until the gel structure was broken, generating stress–strain curves, from which the breaking force and breaking distance were obtained. The test parameters were as follows: P/5S spherical stainless-steel probe, test speed of 120 mm/min, trigger force of 0.4 N, and compression distance of 10 mm. Gel strength was calculated according to Equation (2):

Gel strength (g·mm) = breaking distance (mm) × breaking force (g).(2)

#### 4.7.2. Microstructural Analysis

Frozen-stored MP gels were sectioned into blocks (3 mm × 3 mm × 2 mm) and fixed with 2.5% (*v*/*v*) glutaraldehyde at 4 °C for 24 h. After fixation, the specimens were washed three times with a phosphate buffer (0.2 mol/L, pH 7.2) for 15 min each. Dehydration was performed sequentially in ethanol solutions of 50%, 70%, 80%, and 90% (*v*/*v*) for 15 min per step, followed by three rinses in absolute ethanol (10 min each). The dehydrated samples were then vacuum freeze-dried, gold-sputtered, and examined by scanning electron microscopy (SEM).

#### 4.7.3. Water-Holding Capacity (WHC)

WHC was assessed by centrifugation. In brief, 2.0 g of surimi (W_1_, g) was weighed, wrapped in double-layer filter paper, and transferred to a 50 mL centrifuge tube, followed by centrifugation at 2000× *g* for 15 min at 4 °C. The surimi was immediately removed and reweighed (W_2_, g). WHC was calculated using Equation (3):

(3) $$WHC\left(\%\right)=\frac{W_{2}}{W_{1}}\times 100\%.$$

#### 4.7.4. Water Distribution

Water distribution was characterized by LF-NMR (NMI20-15; Niumag, Suzhou, China) following Zhang et al. [28]. The instrument operated at 0.47 T with a proton resonance frequency of 20 MHz. Approximately 8.00 ± 0.05 g of surimi was packed into 18 mm (i.d.) cylindrical NMR tubes. T_2_ relaxation was recorded using a Carr–Purcell–Meiboom–Gill (CPMG) sequence with an echo time of 1 ms and a repetition time of 2 s. The relaxation time distribution was obtained by processing the data using the CONTIN regularization algorithm. All measurements were performed at 25 °C.

#### 4.7.5. TBARS

The TBARS value was measured according to Luo et al. [53]. Five grams of surimi was extracted with 15 mL of 7.5% (*w*/*v*) trichloroacetic acid containing 0.1% butylated hydroxyanisole and 0.1% ethylenediaminetetraacetic acid. After filtration, 2.5 mL of the extract was mixed with an equal volume of 0.02 mol/L thiobarbituric acid and heated in a boiling water bath for 40 min. The mixture was then cooled, partitioned with chloroform, and centrifuged at 2000× *g* for 10 min at 2 °C. The absorbance of the upper phase was measured at 532 nm using a Cary 4000 spectrophotometer (Agilent Technologies, Santa Clara, CA, USA).

#### 4.7.6. Color

Color parameters were measured using a spectrophotometer (CM-5; Konica Minolta, Japan). The instrument was calibrated with a white reference plate prior to analysis. Redness (a*), yellowness (b*) and lightness (L*) were recorded, and whiteness (W) was calculated using Equation (4):

(4) $$W=100-[\sqrt{{\left(100-L*\right)}^{2}+{a*}^{2}+{b*}^{2}}].$$

### 4.8. Statistical Analyses

All assays were conducted in triplicate, and statistical analyses were carried out using SPSS 21.0 (IBM Corp., North Castle, NY, USA). One-way ANOVA was applied to evaluate differences among groups, and means were compared using Duncan’s multiple range test (*p* < 0.05). Figures were prepared with Origin 2025 (OriginLab Corporation, Northampton, MA, USA).

## Figures and Tables

**Table 1:** Changes in the secondary structure of MPs during freeze–thaw cycles.

Treatment Groups	FT	Random Coil (%)	α-Helix (%)	β-Turn (%)	β-Sheet (%)
CK	0	20.67 ± 0.13 ^Ac^	24.54 ± 0.07 ^Aa^	28.77 ± 0.02 ^Ba^	25.99 ± 0.01 ^Ad^
	1	26.60 ± 0.00 ^Aa^	21.37 ± 0.00 ^Ab^	16.24 ± 0.00 ^Bb^	35.77 ± 0.00 ^Ac^
	3	26.71 ± 0.28 ^Aa^	20.95 ± 0.01 ^Ab^	15.54 ± 0.38 ^Bb^	39.79 ± 0.11 ^Ab^
	5	25.62 ± 0.00 ^Ab^	18.89 ± 0.00 ^Bc^	12.81 ± 0.00 ^Bc^	42.66 ± 0.00 ^Aa^
FVP	0	20.17 ± 0.19 ^Ab^	25.45 ± 1.50 ^Aa^	30.83 ± 0.25 ^Aa^	23.53 ± 1.95 ^Bc^
	1	20.47 ± 0.10 ^Bb^	22.64 ± 0.84 ^Ab^	28.81 ± 0.21 ^Ab^	28.05 ± 0.52 ^Bb^
	3	26.06 ± 0.35 ^Aa^	20.42 ± 0.94 ^Ac^	17.26 ± 1.31 ^Ac^	36.24 ± 0.01 ^Bab^
	5	26.71 ± 0.63 ^Aa^	20.43 ± 0.32 ^Ac^	15.04 ± 2.73 ^Ad^	37.80 ± 2.41 ^Aa^

Values are expressed as mean ± standard deviation (n = 3). Different uppercase letters indicate significant differences (*p* < 0.05) among treatments at the same freeze–thaw cycle, while different lowercase letters indicate significant differences (*p* < 0.05) among freeze–thaw cycles within the same treatment.

**Table 2:** Changes in the area ratio of P_2i_ in surimi during freeze–thaw cycles (FT).

Treatment Groups	FT	P_21_	P_22_	P_23_
CK	0	2.11 ± 0.79 ^Aa^	97.63 ± 0.81 ^Aa^	0.17 ± 0.15 ^Ac^
	1	2.26 ± 0.57 ^Aa^	97.52 ± 0.57 ^Aa^	0.21 ± 0.01 ^Ab^
	3	2.52 ± 0.58 ^Aa^	97.23 ± 0.59 ^Aa^	0.25 ± 0.00 ^Ab^
	5	2.42 ± 0.54 ^Aa^	97.27 ± 0.54 ^Aa^	0.31 ± 0.02 ^Aa^
FVP	0	2.12 ± 0.33 ^Aa^	97.87 ± 0.33 ^Aa^	0 ± 0.00 ^Ba^
	1	2.46 ± 0.80 ^Aa^	97.53 ± 0.80 ^Aa^	0 ± 0.00 ^Ba^
	3	2.44 ± 0.79 ^Aa^	97.55 ± 0.79 ^Aa^	0 ± 0.00 ^Ba^
	5	2.51 ± 0.65 ^Aa^	97.48 ± 0.65 ^Aa^	0 ± 0.00 ^Ba^

Values are expressed as mean ± standard deviation (n = 3). Different uppercase letters indicate significant differences (*p* < 0.05) among treatments at the same freeze–thaw cycle, while different lowercase letters indicate significant differences (*p* < 0.05) among freeze–thaw cycles within the same treatment.

**Table 3:** Effect of FVP on color of surimi during freeze–thaw cycles (FT).

FT	Treatment Groups	L*	a*	b*	Whiteness
0	CK	59.17 ± 1.48 ^Aa^	1.35 ± 0.16 ^Ba^	13.08 ± 0.79 ^Ba^	57.10 ± 1.59 ^Aa^
	FVP	53.22 ± 0.85 ^Ba^	2.77 ± 0.24 ^Ab^	16.61 ± 0.45 ^Aa^	50.28 ± 0.90 ^Ba^
1	CK	54.01 ± 0.55 ^Ab^	1.36 ± 0.12 ^Ba^	11.84 ± 0.47 ^Bb^	52.49 ± 0.62 ^Ab^
	FVP	48.73 ± 0.87 ^Bb^	3.45 ± 0.18 ^Aa^	15.56 ± 1.30 ^Aa^	46.29 ± 0.85 ^Bb^
3	CK	54.06 ± 0.38 ^Ab^	1.39 ± 0.16 ^Ba^	12.42 ± 0.42 ^Bab^	52.39 ± 0.41 ^Ab^
	FVP	48.20 ± 1.68 ^Bb^	2.89 ± 0.49 ^Aab^	16.01 ± 1.13 ^Aa^	45.70 ± 1.93 ^Bb^
5	CK	51.67 ± 0.63 ^Ac^	1.59 ± 0.23 ^Ba^	12.00 ± 0.72 ^Bb^	50.17 ± 0.77 ^Ac^
	FVP	48.53 ± 1.14 ^Bb^	3.42 ± 0.42 ^Aa^	16.34 ± 0.74 ^Aa^	45.88 ± 1.28 ^Bb^

Values are expressed as mean ± standard deviation (n = 3). Different uppercase letters indicate significant differences (*p* < 0.05) among treatments at the same freeze–thaw cycle, while different lowercase letters indicate significant differences (*p* < 0.05) among freeze–thaw cycles within the same treatment.

## Data Availability

The data presented in this study are available in the article.

## Supplementary Materials

The following supporting information can be downloaded at https://www.mdpi.com/article/10.3390/gels12040285/s1, Figure S1: Curve-fitting analysis of the amide I region (1600–1700 cm^−1^); Figure S2: FTIR spectra of MPs from the CK and FVP groups in the wavenumber range of 500–4000 cm^−1^; Table S1: Variations in ΔL*, Δa*, Δb*, and total color difference (ΔE*) of surimi samples during freeze-thaw cycles.
