# Supplementary material for: Root-Derived Flammulina velutipes Polysaccharides Improve Myofibrillar Protein Stability and Maintain Catfish Surimi Quality During Freeze–Thaw Cycling
Source: Gels. 2026 Mar 28;12(4):285. doi: 10.3390/gels12040285 (PMC13115371; doi:10.3390/gels12040285)
Supplement: Supplementary file 1 [file gels-12-00285-s001.zip › gels-4169585-supplementary.pdf]

**Supplementary Figure S1.** Curve-fitting analysis of the amide I region (1600-1700 $cm^{-1}$)

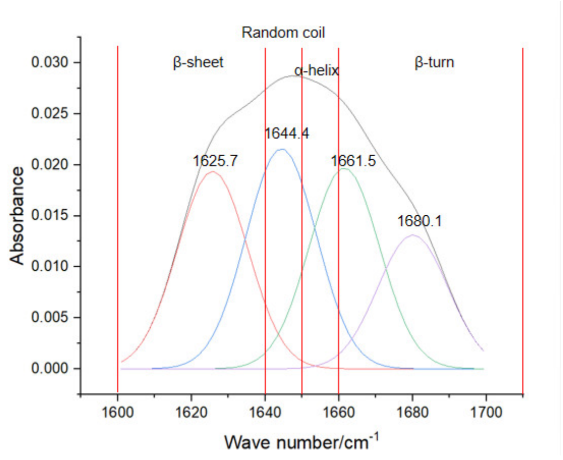


**Supplementary Figure S2.** FTIR spectra of MPs from the CK and FVP groups in the wavenumber range of 500-4000 $cm^{-1}$.

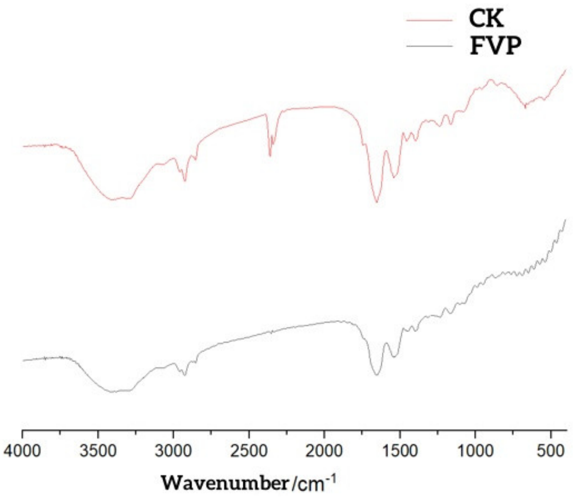


**Supplementary Table S1.** Variations in ΔL*, Δa*, Δb*, and total color difference (ΔE*) of surimi samples during freeze-thaw cycles.

| FT | Treatment groups | ΔL* | Δa* | Δb* | ΔE* |
|---|---|---|---|---|---|
| 1 | CK | $5.16\pm1.54^{Aa}$ | $0.18\pm0.17^{Ba}$ | $1.25\pm0.82^{Aa}$ | $5.43\pm1.26^{Aa}$ |
| | FVP | $4.49\pm0.87^{Aa}$ | $0.68\pm0.26^{Aa}$ | $1.16\pm1.26^{Aa}$ | $4.84\pm0.82^{Aa}$ |
| 3 | CK | $0.50\pm0.44^{Ac}$ | $0.23\pm0.09^{Ba}$ | $0.84\pm0.52^{Aa}$ | $1.11\pm0.44^{Ac}$ |
| | FVP | $1.16\pm1.64^{Ab}$ | $0.69\pm0.36^{Aa}$ | $1.29\pm0.97^{Aa}$ | $2.14\pm1.55^{Ab}$ |

| | | | | | |
|---|---|---|---|---|---|
| **5** | CK | 2.40±0.51$^{Ab}$ | 0.21±0.18$^{Ba}$ | 0.82±0.57$^{Aa}$ | 2.61±0.44$^{Ab}$ |
| | FVP | 1.4 ±0.89$^{Bb}$ | 0.75±0.40$^{Aa}$ | 0.94±0.94$^{Aa}$ | 2.03±1.06$^{Ab}$ |

Values are expressed as mean ± standard deviation (n = 3). Different uppercase letters indicate significant differences ($p < 0.05$) among treatments at the same freeze-thaw cycle, while different lowercase letters indicate significant differences ($p < 0.05$) among freeze-thaw cycles within the same treatment.
